# Characterization and Properties of Water-Blown Rigid Polyurethane Foams Reinforced with Silane-Modified Nanosepiolites Functionalized with Graphite

**DOI:** 10.3390/ma15010381

**Published:** 2022-01-05

**Authors:** Mercedes Santiago-Calvo, María Carracedo-Pérez, María Luisa Puertas, Antonio Esteban-Cubillo, Julio Santaren, Fernando Villafañe, Miguel-Ángel Rodríguez-Pérez

**Affiliations:** 1 Cellular Materials Laboratory (CellMat), Condensed Matter Physics Department, Faculty of Science, Campus Miguel Delibes, University of Valladolid, Paseo de Belén 7, 47011 Valladolid, Spain; maria.carracedo@alumnos.uva.es (M.C.-P.); marrod@fmc.uva.es (M.-Á.R.-P.); 2 Research and Development Department, Tolsa S. A., Carretera de Madrid a Rivas Jarama, 35, 28031 Madrid, Spain; mpuertas@tolsa.com (M.L.P.); aesteban@tolsa.com (A.E.-C.); jsantaren@tolsa.com (J.S.); 3 GIR MIOMeT-IU Cinquima-Química Inorgánica, Faculty of Science, Campus Miguel Delibes, University of Valladolid, Paseo de Belén 7, 47011 Valladolid, Spain; fernando.villafane@uva.es

**Keywords:** flame retardant, polyurethane foam, thermal stability, nanosepiolites, graphite

## Abstract

In the present study, a promising flame retardant consisting of 80 wt% silane-modified nanosepiolites functionalized with 20 wt% graphite (SFG) is used to obtain a synergistic effect principally focussed on the thermal stability of water-blown rigid polyurethane (RPU) foams. Density, microcellular structure, thermal stability and thermal conductivity are examined for RPU foams reinforced with different contents of SFG (0, as reference material, 2, 4 and 6 wt%). The sample with 6 wt% SFG presents a slightly thermal stability improvement, although its cellular structure is deteriorated in comparison with the reference material. Furthermore, the influence of SFG particles on chemical reactions during the foaming process is studied by FTIR spectroscopy. The information obtained from the chemical reactions and from isocyanate consumption is used to optimize the formulation of the foam with 6 wt% SFG. Additionally, in order to determine the effects of functionalization on SFG, foams containing only silane-modified nanosepiolites, only graphite, or silane-modified nanosepiolites and graphite added separately are studied here as well. In conclusion, the inclusion of SFG in RPU foams allows the best performance to be achieved.

## 1. Introduction

The distinctive combination of low weight, low thermal conductivity and good mechanical properties makes rigid polyurethane (RPU) foam an indispensable thermal insulating material in buildings, insulated transport, refrigerators and pipelines, among other applications [1]. Nevertheless, in case of fire these foams have huge drawbacks such as high flammability, fast flame-spreading rate and formation of toxic gases. For these reasons, many studies have investigated enhancing the flame resistance of PU foams to meet the strict requirements of flame retardancy, particularly in the construction sector.

Given the above, diverse kinds of flame retardants (FRs) are currently being tested to enhance the thermal stability of PU materials, including FRs containing halogens, FRs without halogens (phosphonium salts, layered silicates, ammonium polyphosphates or expandable graphite), and reactive FRs (polyols containing nitrogen-, phosphorous- or silicon-compounds) [2,3]. Among these, the halogenated FRs, which cover all organic compounds containing in their composition the elements of group 17, have been extensively used for many years due to their high efficiency in flame retardancy. However, they have a major drawback in that a large amount of toxic polluting gases are generated during their combustion. For this reason, the halogen-free FRs were developed from compounds based on phosphorus, nitrogen or silicon as substitutes for halogen-based FRs [4,5,6,7].

Graphite is a very popular halogen-free FR used in PU foams because it is economical and because effective flame retardancy is reached with low amounts. Graphite is one of the allotropic forms of carbon. The structure of graphite is based on stacked graphene sheets [8]. Its fire-retardant properties are based on the charred layer formed during combustion, which acts as a thermal insulator prior to the degradation of the polymeric matrix. This effect is due to the small air spaces between individual layers of graphite [9]. Many investigations show that the use of graphite significantly enhances fire resistance [10,11,12,13,14,15].

Reports on the use of sepiolite as a halogen-free FR of PU foams are scarce [16,17,18,19,20,21,22]. Sepiolite is a natural clay mineral of the structural family known as the 2:1 phyllosilicates, with (Si_12_Mg_8_O_30_)(OH)_4_(OH_2_)_4_·8H_2_O as theoretical unit cell formula [23]. It exhibits a microfibrous morphology formed by two layers of tetrahedral silica and a central layer of magnesium oxide-hydroxide. These components have little affinity for low polarity polymers due to the existence of silanol groups (Si–OH) on the sepiolite surface, which are very polar and hydrophilic [24,25,26]. Thus, the compatibility between the sepiolite and the polymer must be improved by modifying the sepiolite surface with apolar organic chemicals. Regarding flame-retardant properties, sepiolites act as a thermal insulator because its crystal structure is not modified; consequently, these particles remain in the char, increasing the residue mass [24,27]. In addition, sepiolites act as transport barriers for the volatile products generated during decomposition [24,27].

In this study, a promising halogen-free FR consisting of 80 wt% silane-modified nanosepiolites functionalized with 20 wt% graphite (SFG) was incorporated into water blown RPU foams. This work was designed in the expectation that the synergistic effects of nanosepiolites and graphite together might be effective for enhancing the thermal stability of PU materials.

## 2. Experimental

### 2.1. Raw Materials

Alcupol R4520, a high functionality polyether polyol from Repsol SA, was used to manufacture the foams under study. The characteristics of the polyol used were functionality 4.5, 455 mg KOH/g of hydroxyl and 5250 mPa·s viscosity. IsoPMDI 92140, a polymeric diphenylmethane diisocyanate from BASF Poliuretanos Iberia S.A, was employed to manufacture the foams. The characteristics of the isocyanate were functionality 2.7, 31.5% NCO content, and 210 mPa·s viscosity. TEGOAMIN^®^ DMCHA from Evonik based on N,N-dimethylcyclohexylamine, was employed as a blowing and gelling catalyst. TEGOSTAB^®^ B 8522 from Evonik based on a non-hydrolysable poly-ether-polydimethyl-siloxane–stabilizer was employed as a surfactant. The chemical blowing agent was distilled water.

Silane-modified nanosepiolites functionalized with graphite (SFG) supplied by Tolsa SA were used as flame retardants (FR). According to the supplier, the SFG particles contained 80 wt% of silane-modified nanosepiolites (S), with length between 1 and 2 µm and diameter between 20 and 30 nm, and 20 wt% of graphite (G) from Sigma Aldrich, with a diameter below 20 µm. The nanosepiolites were modified with silane groups to enhance the affinity between the particle and the polyurethane matrix. The particles were dried under vacuum at 90 °C overnight before the foams were manufactured.

### 2.2. Synthesis of Rigid Polyurethane Foams Reinforced with Silane-Modified Nanosepiolites Functionalized with Graphite (SFG)

In the first part of this study, four RPU foams with different weight percentages (wt%, respect to the total mass of the foam) of FR were examined: the reference material with 0 wt% SFG particles, and the materials with 2 wt% SFG, 4 wt% SFG and 6 wt% SFG. The formulation used and the SFG contents are summarised in Table 1. Moreover, samples with SFG contents above 6 wt%, particularly samples containing 8 wt% SFG and 10 wt% SFG, were prepared following the same foam formulation shown in Table 1. However, the samples with higher particles contents could not be characterized because these materials did not foam well, presenting a highly deteriorated cellular structure and low expansion.

As described below (Section 3.2.2), the main result of the first part of the study is the higher thermal stability of the foam containing 6 wt% SFG. Therefore, in the second part of this study, the formulation of this foam was optimized by changing the amine catalyst (DMCHA, N,N-dimethylcyclohexylamine) from 1 part per weight (ppw) to 1.25 and 1.5 (Table 2).

In the third and final part of this study (Section 3.3), the material with 6 wt% of functionalized particles (SFG containing 80% nanosepiolites and 20% graphite, and thus 4.8 wt% and 1.2 wt%, respectively, in the foam) and the optimum amount of catalyst (1.5 ppw DMCHA) was compared to other foams which contained (i) only nanosepiolites, (ii) only graphite and nanosepiolites, or (iii) only graphite, added in a different way. The objective here was to corroborate that the functionalization of the SFG particles is the determining factor of their best thermal stability. The studied foams are summarized in Table 3.

**Figure 1:**
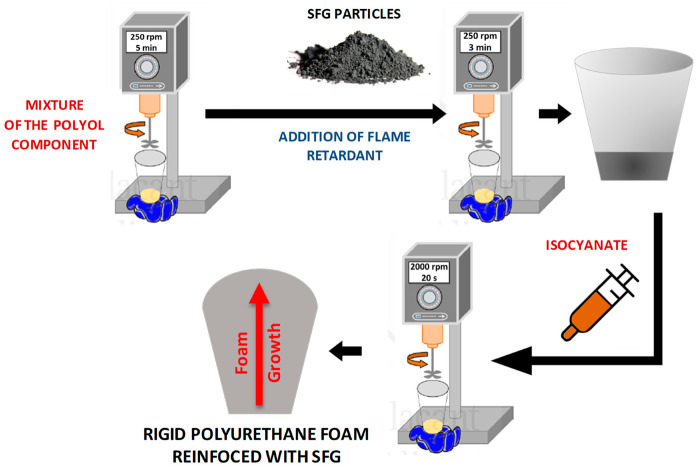
Schematic diagram of practical preparation of RPU foams reinforced with SFG.

The foams were produced following the procedure detailed below. Initially, the polyol component was prepared by mixing the polyol, the surfactant, the catalyst and the blowing agent at 250 rpm for 5 min using an overhead stirrer from IKA (EUROSTAR 60 control) with a Lenart disc stirrer of 50 mm diameter. After that, the FR was dispersed in the polyol at low shear stress rates of 250 rpm for 3 min. Subsequently, the isocyanate and the polyol containing the FR were mixed in a plastic cup at 2000 rpm for 20 s, using the mechanical stirrer described above in order to promote the foaming process. Foams with an almost cylindrical (specifically, truncated cone) shape of approximately 20 cm of height and 10 cm average diameter were obtained. After foaming, the materials were cured at room temperature for the 24 h following their manufacture. The scheme for the foam preparation procedure is illustrated in Figure 1.

### 2.3. Characterization of Silane-Modified Nanosepiolites Functionalized with Graphite (SFG)

Three methods were used for the characterization of the new FR based on SFG. Scanning electron microscopy (SEM) was performed to characterize the particle morphology using a FlexSEM 1000 microscope from Hitachi. Fourier transform infrared (FTIR) spectroscopy was used to identify the surface groups of the particles using a Bruker Tensor 27 spectrometer in the attenuated total reflectance (ATR) mode. Thermogravimetric analysis (TGA) was used to evaluate the thermal behaviour of the SFG particles using a Mettler Toledo TGA/SDTA 851. The TGA thermogram was carried out from 50 to 600 °C at a heating rate of 10 °C/min and under inert atmosphere (N_2_). 

### 2.4. Characterization of RPU Foams

The density of three cylindrical samples (with dimensions of 30 mm of diameter and 30 mm of height) for each material was measured according ASTM D1622/D1622M-14 [28].

After measuring the density of the samples, open cell content (OC) was determined according to ASTM D6226-10 by using a gas pycnometer, Accupyc II 1340 from Micromeritics [29].

The cellular structure of the foams was observed by Scanning electron microscopy (SEM). SEM micrographs of each material were acquired by a FlexSEM 1000 microscope from Hitachi. To that end, a smooth surface of cured foams was covered with a gold monolayer and the growth direction was observed by SEM after vacuum. After that, an image analysis method [30] was used to calculate the main parameters of the microstructure, such as average cell size in 3D (Φ3D), anisotropy ratio (AR) and normalized standard deviation (NSD). The latter is the ratio between the standard deviation (SD) of the cell size distribution and the Φ3D, and gives information about the homogeneity of the cell size distribution; thus, homogeneous cell distributions present small values of this parameter (in fact, much smaller than 1 for this ratio). To calculate these cellular structure parameters, this analysis was made by using at least 100 cells of different areas for each material.

TGA was employed to investigate the thermal stability of the foams under study and was carried out using a Mettler Toledo TGA/SDTA 851 under inert atmosphere (N_2_); 10 mg Samples were heated from 50 to 600 °C at a heating rate of 10 °C/min. Two TGAs were carried out for each sample, which gave similar results, and the average of the TGAs is represented here.

Viscosity measurements of neat polyol and polyol/SFG mixtures were carried out at 25 °C using a ROTAVISC lo-vi Complete viscosimeter from IKA.

Thermal conductivity was measured by a hot disk thermal constant analyzer (Hot-Disk TPS 2500S) at room temperature using two cylindrical samples (with dimensions of 30 mm of diameter and 30 mm of height) for each material. To obtain the thermal measurements, a disk-shaped TPS sensor (Hot Disk, Gothenburg, Sweden) with a radius of 3.189 mm was located between the two samples in contact with the xy plane, perpendicular to the growth plane. A total of five measurements were obtained for each material once the foam reached a stationary state, approximately two weeks after production, in which the foam cells only contained atmospheric air because all of the carbon dioxide formed during the foam production had diffused out of the cells [31].

### 2.5. Study of Foaming Kinetics by FTIR Spectroscopy

**Figure 2:**
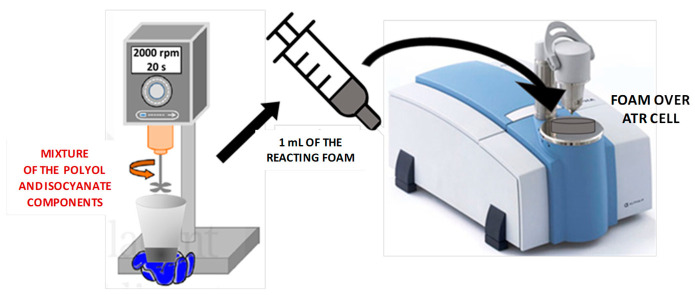
Scheme of the procedure followed to obtain the in situ variation of FTIR spectra versus time.

A Bruker ALPHA spectrometer in the attenuated total reflectance (ATR) mode was used to acquire FTIR spectra with time for the foams under study. Before starting to take FTIR spectra over time, a background spectrum was acquired in order to subtract it from each FTIR spectrum. As shown in Figure 2, 1 mL of reacting foam was deposited on the ATR cell immediately after the stirring process to acquire FTIR spectra. The time zero of the reaction kinetics was taken when the isocyanate was added to the polyol and the first spectrum was acquired at around 50 s. The parameters selected to acquire the FTIR spectra were 16 scans, 4 cm^−1^ of resolution, a range of wavenumber from 4000 to 400 cm^−1^ and a temperature of 70 °C to reproduce the exothermic foaming process of an RPU foam. Three experiments were taken for each material and 60 spectra were taken for each 30 min experiment.

Once the spectra were obtained, a baseline correction was performed to correct intensity changes at low frequencies. After that, the concentration or density modifications during the foaming process were corrected using the asymmetric CH stretching band at 2972 cm^−1^ as an internal reference, due to its concentration remaining constant during the foam formation [32,33,34,35].

In situ FTIR spectroscopy is a suitable technique to monitor the isocyanate conversion and product generation. First, the consumption of isocyanate during foam formation was measured by determining the area decay of the isocyanate absorption band at 2270 cm^−1^. In addition, a deconvolution of the carbonyl region was followed as indicated in the literature [36,37,38] to quantify the amount of reaction products. The FTIR spectra selected were those obtained after 50, 80, 110, 170, 300, 600, 900, 1200 and 1800 s.

## 3. Results and Discussion

### 3.1. Characterization of SFG

A complete characterization of the new FR was carried out in order to better comprehend the events happening during the foaming, for instance the interaction of the SFG particles with the PU matrix and with the reactions, as well as any possible nucleating effect. Therefore, the first step is the characterization of the SFGs used.

**Figure 3:**
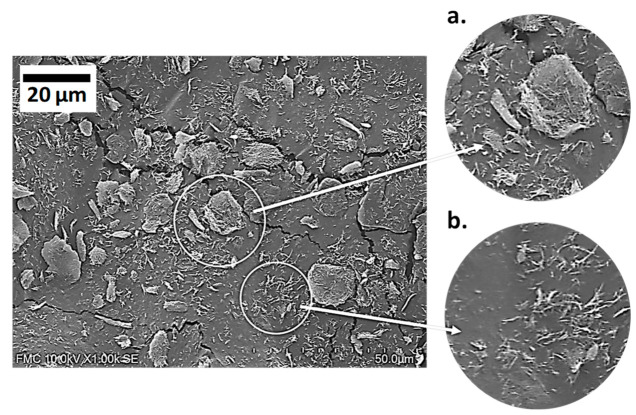
Left: SEM micrograph of SFG particles. Right: Detailed views of (**a**) nanosepiolites functionalized with graphite; (**b**) nanosepiolites.

The morphology of the SFG particles was observed by SEM (Figure 3), confirming the particle sizes supplied by the manufacturer and the functionalization of the nanosepiolites with graphite. The nanofibers detected in Figure 3b are nanosepiolites, with an average particle length varying between 1 and 2 µm and a diameter in the nanometer range of approximately 20 and 30 nm. Graphite particles are the largest, showing particle sizes below 20 µm. The nanosepiolites deposited on the graphite surface can be seen in Figure 3a, which verifies the functionalization of the nanosepiolites.

**Figure 4:**
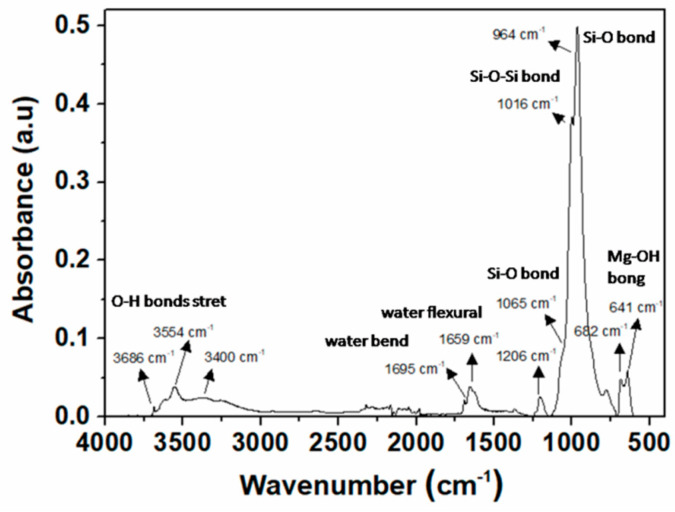
FTIR spectrum of a SFG particle.

The composition of SFG particles was supported by FTIR spectroscopy (Figure 4). As expected, the characteristic bands of SFG particles are very similar to those of sepiolites. The bands in the range 4000–3000 cm^−1^ are associated with O-H bond stretching vibrations: vibrations of the Mg–OH groups at 3686 cm^−1^, vibrations of coordinated water at 3554 cm^−1^ and vibrations of zeolitic water at 3400 cm^−1^, supporting the presence of water in the channels of the sepiolite. The band at 1695 cm^−1^ coincides with the bending movement of the water adsorbed on the surface of the sepiolite, whereas the band at 1659 cm^−1^ is attributed to the flexural vibrations of zeolitic water. The 1200–400 cm^−1^ range is characteristic of silicates: an absorption centered at 1016 cm^−1^ due to the Si-O-Si vibration, other bands at 1206, 1065 (shoulder) and 964 cm^−1^ due to Si–O bonds, and bands at 682 and 641 cm^−1^ coinciding with Mg-OH bond vibrations [39,40,41].

**Figure 5:**
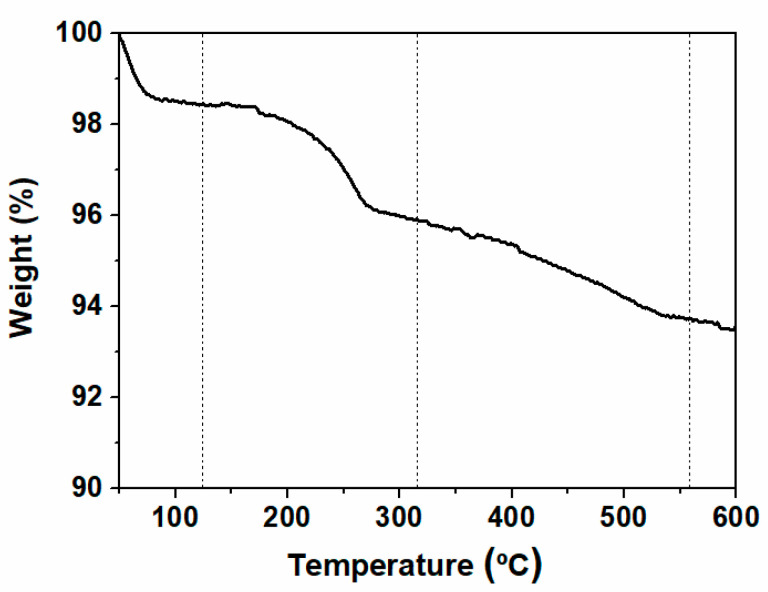
TGA of the SFG particles.

The thermal stability of the SFG particles was evaluated by TGA. The thermogram of SFG representing the percentage of residue (93–100%) versus the temperature (50–600 °C) is depicted in Figure 5. These results show that the SFG particles are composed of graphite and nanosepiolites modified with silane groups, as indicated above. Previous investigations [42] concluded that graphite decomposition does not occur in the temperature range or in the atmosphere in which the study was carried out (N_2_). Therefore, the loss of mass from the SFG particles should exclusively be due to the degradation of the nanosepiolites modified with silane groups. The three steps clearly displayed in the thermogram of the SFG particles are very similar to those observed in the thermograms of sepiolites [39,41,43]. The mass loss in the SFG particles and that in sepiolites are analogous, and must therefore be based mainly on the evaporation of the modifier added to the sepiolites (silane groups) as well as on the evaporation of the water contained in the sepiolites: zeolitic water, surface-adsorbed water and coordinated water.

The first weight loss occurs from the beginning of the analysis up to 120 °C, and coincides with the evaporation of both zeolitic water and the water adsorbed on the surface of the particles. The second degradation, located between 120 and 300 °C, is due to the evaporation of a part of the coordinated water in the sepiolites and leaves a residue very close to 96% of the initial weight. The third stage is due to the evaporation of the remaining coordinated water contained in the sepiolites, and is only completed when reaching 600 °C. This third degradation is lower than the previous two, however more than 2% of the initial material is now being degraded. Finally, a residue of around 93% is obtained at 600 °C, that is, less than 7% of the initial mass is lost, of which more than 4% is due to water. 

### 3.2. Study of Rigid Polyurethane Foams with SFG

#### 3.2.1. Density and Cellular Structure Characterization

The density and cellular structure affect the final properties and applications of RPU foams, and therefore are very relevant. In particular, the densities and main characteristics of the cellular structure for the foams containing SFG (0, 2, 4 and 6 wt%), such as open cell content (OC), average cell size in 3D (Φ3D), and anisotropy ratio (AR) are collected in Table 4. Moreover, the viscosities of pure polyol and the polyol/SFG dispersion used for each foam are shown in Table 5.

The density values are displayed in the second column of Table 4. In general, the density increases with the amount of particles added. The density of the foams with 2 wt% and 4 wt% SFG increases around 2 kg/m^3^ compared to the reference foam, whereas the density of the foam containing 6 wt% SFG increases by around 4 kg/m^3^. This relationship between the density and the amount of SFG particles could be due to the increase in viscosity when the SFG particles are incorporated (Table 5), which could hinder the growth of the foam.

In addition, OC (3rd column in Table 4) shows a clear trend, as it increases when the amount of SFG increases. The OC of the foam with a lower particle content (2 wt%) slightly increases compared to that of the reference foam. However, both foams have a low OC, and they can be considered closed cells. Furthermore, the samples with higher amounts of SFG (4 and 6 wt%) show a large increase in OC in comparison with that of the unfilled foam. In these cases, the increase in the amount of SFG could favour particle agglomerations, which would promote the opening of the cell walls, and thus cell degeneration. This effect has been observed previously in our group studying RPU foams with high contents of nanoclays and nanosilicas [38].

**Figure 6:**
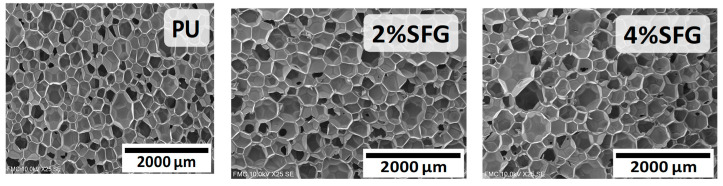

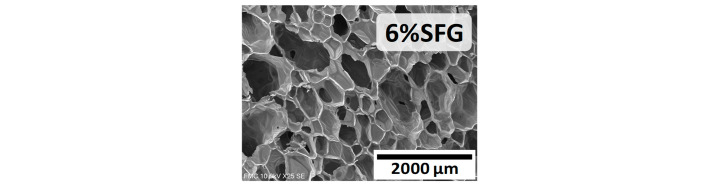
SEM micrographs in the growing plane of the RPU foams: Unfilled foam and foams with different SFG contents.

The three latter columns in Table 4 are discussed with the help of the micrographs obtained by SEM in the growing direction of the manufactured foams, shown in Figure 6. These micrographs provide quantitative information about cell size, the homogeneity of the cell size distribution, and other characteristic parameters of the cellular structure such as the AR (Table 4). The cell size (Φ3D) rises with the content of SFG particles. Thus, the materials with the higher content of SFG (6 wt%) present the largest cell size (around 46% more than that of the reference foam). Regarding the homogeneity of the cellular structure, the reference foam and those with 2 and 4 wt% SFG show a very homogeneous cellular structure (low and similar values of NSD). Conversely, the foam with the higher content has a heterogeneous cellular structure, as it presents a higher NSD value. Finally, all foams can be considered isotropic, as they have an AR close to 1 (last column in Table 4).

#### 3.2.2. Thermal Properties by Thermogravimetric Analysis

**Figure 7:**
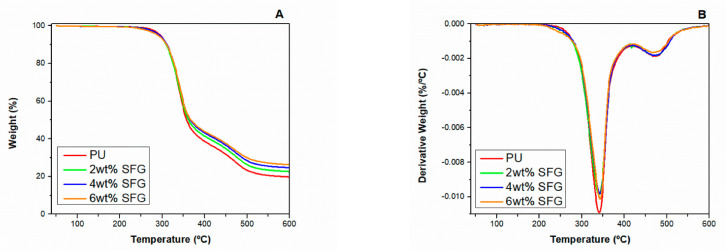
(**A**) TGA and (**B**) DTG curves for RPU foams containing SFG from 0 to 6 wt%.

TGA is one of the most relevant kinds of analysis for evaluating the thermal properties of PU foams. Figure 7 shows the influence of SFG on the thermal stability of the RPU foams in N_2_ atmosphere by means of TGA graphs and the corresponding first derivative of the TGA curve, DTG. Both the reference foam and the foams containing SFG show a similar degradation curve shape for each sample, although the values are slightly different. Table 6 collects the thermal parameters obtained: the initial decomposition temperature (T5, that is the temperature at 5% weight loss), the temperature at maximum weight loss rate in the first step (Tmax1, denoted as the peak value from the DTG curves), the maximum loss weight rate in the first step (DGTmax1, denoted as the peak value from the DTG curves), the temperature at maximum weight loss rate in the second step (Tmax2, denoted as the peak value from the DTG curves), the maximum loss weight rate in the second step (DGTmax2, denoted as the peak value from the DTG curves, and the remaining residues at the end of the first step and at the end of the second step.

**Scheme 1:**
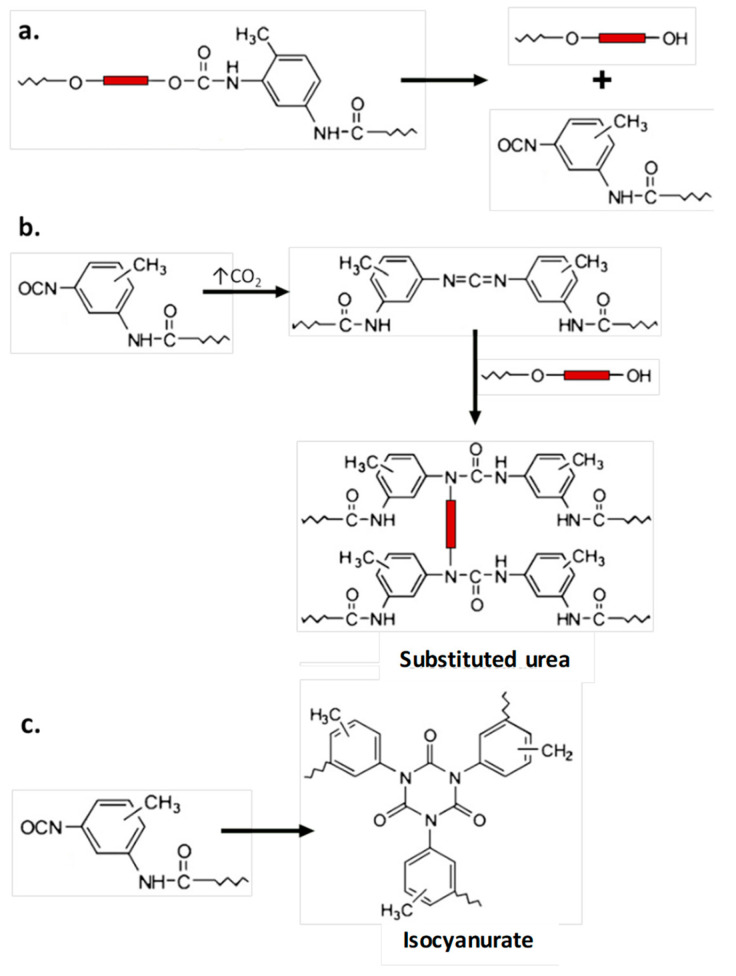
Main reactions involved in the first stage of thermal degradation of PU foams: (**a**) urethane bond scission, (**b**) formation of substituted ureas and (**c**) formation of isocyanurate.

The combined representation of the TGA and DTG curves help to deduce the reactions involved in thermal decomposition and to determine the temperature at which these reactions occur. The thermograms for the different foams present similar curves, with two main losses of mass at around 340 °C and 470 °C. This result, corroborated by the DTGs, can be explained considering that the degradation of PU foams in an N_2_ atmosphere generally occurs following two stages (Scheme 1) [44,45,46]:

- The first stage consists of several degradation processes of the urethane bond to give back to the main precursors, alcohol and isocyanate (Scheme 1a), and to form volatile compounds such as alcohols, CO_2_, CO, aldehydes, amines, etc. (not shown in Scheme 1) [44,45,46,47,48,49]. Complete volatilization of the resulting chain fragments is prevented by dimerization of the isocyanate, which is tremendously reactive, releasing CO_2_ and generating carbodiimides. These carbodiimides react with the alcohol freed from the previous decomposition to give substituted ureas, which are relatively stable [44,45,46] (Scheme 1b). In addition, trimerization of the isocyanate can take place under certain conditions to give very thermally stable isocyanurate rings (Scheme 1c).
- The second stage involves the degradation at high temperatures of the mentioned stable structures (substituted ureas, isocyanurates), to produce volatile products and a small carbonized layer or char (a complex insoluble material) [44,45,46,47,48,49].

TGA results indicate that the T5 of the reference material is 295 °C, and this value slightly decreases to 288 °C as the amount of SFG increases. However, Tmax1 slightly increases for the foams containing SFG, reaching 343 °C for the material with the higher SFG content, whereas the rate of thermal degradation slows down, with SFG reaching lower values of DTGmax1 (Table 6). The most remarkable result is the increase of the percentage of residue when SFG is incorporated, which goes from 36% for the unfilled foam to 41% for the foam containing 6 wt% SFG. A similar behaviour is observed in the second step of degradation. The Tmax2 values reached for the reference foam and for the foam with 6 wt% SFG are very close; however, the rate of thermal degradation is reduced for the material with 6 wt% SFG, as can be observed in Table 6 as a lower value of DTGmax2. Regarding the final residue, the observed behaviour is similar to that detected in the first degradation, as the residue significantly increases when SFG is added, going from ca. 20% for the unfilled foam to 26% for the foam containing 6 wt% SFG. Thus, these results evidence that the thermal stability is slightly enhanced with SFG, especially for the higher content. 

#### 3.2.3. Influence of SFG on Reaction Kinetics

The TGA analysis described above concludes that the SFG particles increase the thermal stability of RPU foam. However, the incorporation of this FR clearly deteriorates the cellular structure and increases the density. However, the properties of the foam can be improved by analysing the influence of SFGs on the RPU foam reaction kinetics in order to correct their effect. For this purpose, in situ FTIR spectroscopy was used to follow the reaction kinetics of RPU foams containing different amounts of SFG. Two variables were studied: the isocyanate consumption, which is estimated by the reduction of the isocyanate asymmetric stretching vibration at 2270 cm^−1^, and the formation of urethane and urea compounds, which are determined by the rise of the carbonyl stretching vibrations in the range of 1610–1760 cm^−1^.

**Figure 8:**
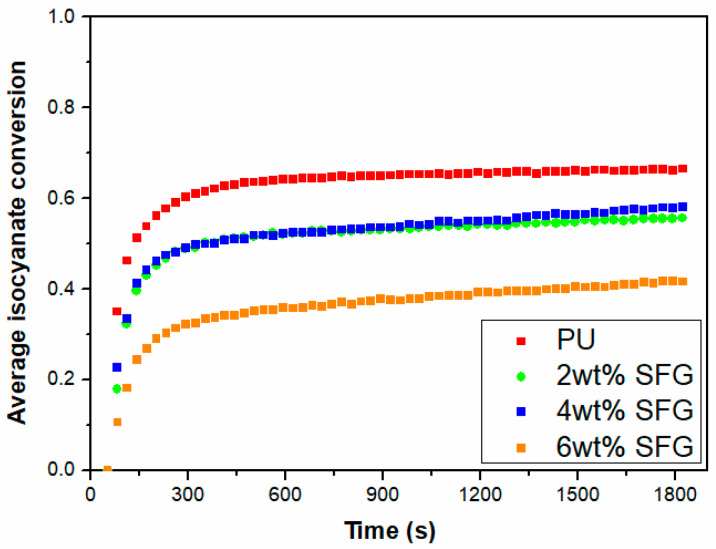
Average isocyanate consumption versus time for RPU materials containing SFG, from 0 to 6 wt%.

Figure 8 shows that the SFG particles reduce the isocyanate consumption during all foaming processes. The highest isocyanate conversion occurs for the reference foam (around 66%), followed by the foams with 2 and 4 wt% SFG, which have very similar conversion (50% and 55%, respectively). The lowest isocyanate conversion (40%) occurs for the foam with 6 wt% SFG. This effect may be due to the increase in viscosity occurring when more particles are present, as can be seen in Table 4, which causes a reduction in the mobility of the reagents and the contact between them, reducing the consumption of isocyanate. In addition, the slopes of the curves in Figure 8 indicate certain conclusions about the reaction rates. The fastest reaction occurs for the reference foam, whereas the curve corresponding to the foam with 6 wt% SFG reaches its maximum value more slowly. The curves corresponding to the foams containing 2 and 4 wt% SFG increase similarly to the reference foam, though reaching lower values of isocyanate conversion. Therefore, it can be concluded that there is an inversely proportional relationship between the reaction rate and the amount of FR in the PU foam.

**Figure 9:**
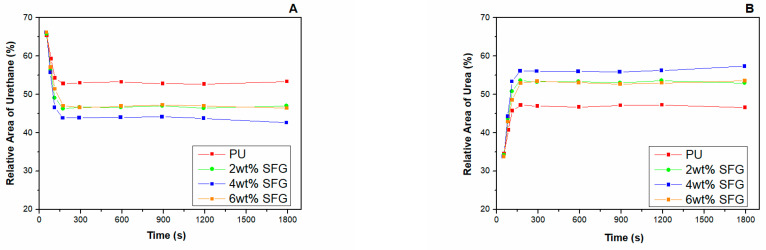
Relative area percentage of the absorbances of urethane (**A**) and urea (**B**) identified in the carbonyl region over time for RPU foams containing SFG.

The carbonyl stretching vibrations in the range of 1610–1760 cm^−1^ provide information about generation of urethanes and ureas. These carbonyl absorptions were monitored and deconvoluted [38], allowing the quantification of urea and urethane by the relative area percentages of ureas and urethanes relative to the whole area of all the carbonyl absorptions. Therefore, the two main reactions (blowing and gelling) may be followed independently with this information. Figure 9 collects the relative area percentages of urethane (Figure 9A) and urea (Figure 9B) vs. time. The graphs clearly show that the percentage of ureas and urethanes remains constant after the first ca. 400 s; that is, the formation reaction of these products does not evolve, thus confirming the results observed for the consumption of isocyanate (Figure 8). Figure 9A,B shows that the formation of ureas and urethanes vary as a function of the amount of SFG. Thus, the lowest percentage of ureas, close to 45%, is obtained for the reference foam, which instead generates the highest percentage of urethanes. The opposite occurs for the foam containing 4 wt% SFG, because it generates 55% of ureas and ca. 40% of urethanes. Foams with 2 and 6 wt% SFG give rise to very similar results. Considering the data in both Figure 8 and Figure 9, the consumed isocyanate in the reference foam is mainly used in the polymerization reaction, as urethanes are the major product, while the presence of SFG particles in the foam tends to promote the products from the foaming reaction (ureas). This could be an effect of the water contained in the SFG, which may be released during foam formation, as the temperature inside the foam reached more than 150 °C [33]; the released water could react with the isocyanate, generating urea and CO_2_. The released water from the SFG FR could be zeolitic water, surface-adsorbed water or coordinated water, as observed in the TGA analysis (Section 3.1). The conclusion obtained from the FTIR results is the decrease of isocyanate conversion and of the polymerization reaction with increasing amounts of SFG.

#### 3.2.4. Optimization for the Polyurethane Foam Formulation Containing 6 wt% SFG

Contemplating all the above results, we planned to modify the foam formulation of the material with 6 wt% SFG, which is the material with the best performance regarding thermal stability, in order to improve its cellular structure and decrease its density. To this end, several RPU foams containing 6 wt% SFG were obtained by varying the amounts of catalyst (1 ppw, contained in the foam described above, as well as 1.25 and 1.5 ppw). The aim of this strategy was to promote a higher isocyanate conversion in the gelling reaction, following the kinetic results previously mentioned. 

Table 7 collects the density values and cellular structure characteristics of the samples containing 6 wt% SFG obtained with different catalyst contents (from 1 to 1.5 ppw). The density data show a slight decrease when the catalyst amount is increased, as the density of the foam obtained with the highest content (1.5 ppw) of catalyst decreases by 3 kg/m^3^ in respect of the foam with a lower content (1 ppw) of catalyst. This may be explained by considering that the consumption of isocyanate is favoured with higher amounts of catalyst; thus, the high degree of polymerization precludes the escape of the gas generated in the foaming reaction. Thus, the expansion efficiency is improved with the increase of the catalyst percentage in the PU formulation. Moreover, the open cell content is slightly reduced with the addition of catalyst, from 17.59% for the sample with 6 wt% SFG and 1 ppw catalyst to 7.89% for the sample with 6 wt% SFG and 1.5 ppw of catalyst. This reduction in open cell content may be related to higher crosslinking promoted by increasing the catalyst.

**Figure 10:**
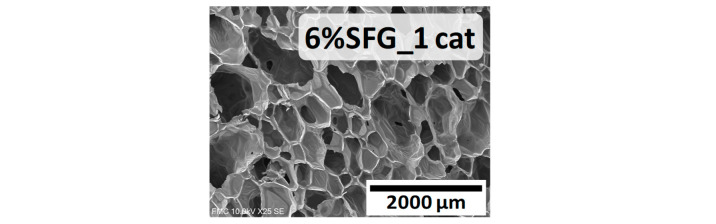

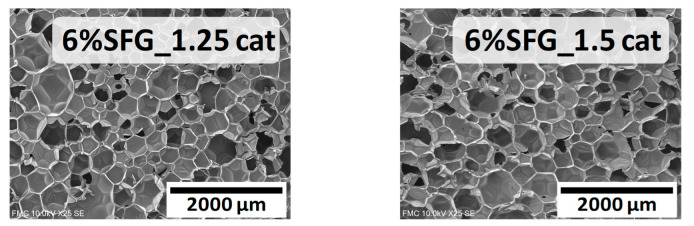
SEM micrographs in the growing plane of the RPU foams containing 6 wt% SFG and different DMCHA catalyst contents (1, 1.25 and 1.5 ppw).

On the other hand, Table 7 and Figure 10 show how an increase in the amount of DMCHA catalyst generates a clear cell size reduction. The cell sizes of the foams obtained with 1.25 and 1.5 ppw catalyst are around 400 µm, which represents a reduction of 20% with respect to the cell size of the foam containing 1 ppw catalyst (around 500 µm). Additionally, Table 7 shows that the samples obtained with higher amounts of catalyst content are more homogeneous than the sample obtained with 1 ppw catalyst, as the value of NSD is reduced. Additionally, the higher amounts of catalyst reduce the anisotropy, with the sample obtained with 1.5 ppw catalyst having the lowest value.

These results indicate that the better-performing formulation is the PU obtained with 1.5 ppw of DMCHA catalyst, as it leads to a final material with significant reductions in density, open cell content, cell size and anisotropy.

### 3.3. Validation of Improvement in Thermal Stability of the Polyurethane Foam Containing 6 wt% of SFG

**Figure 11:**
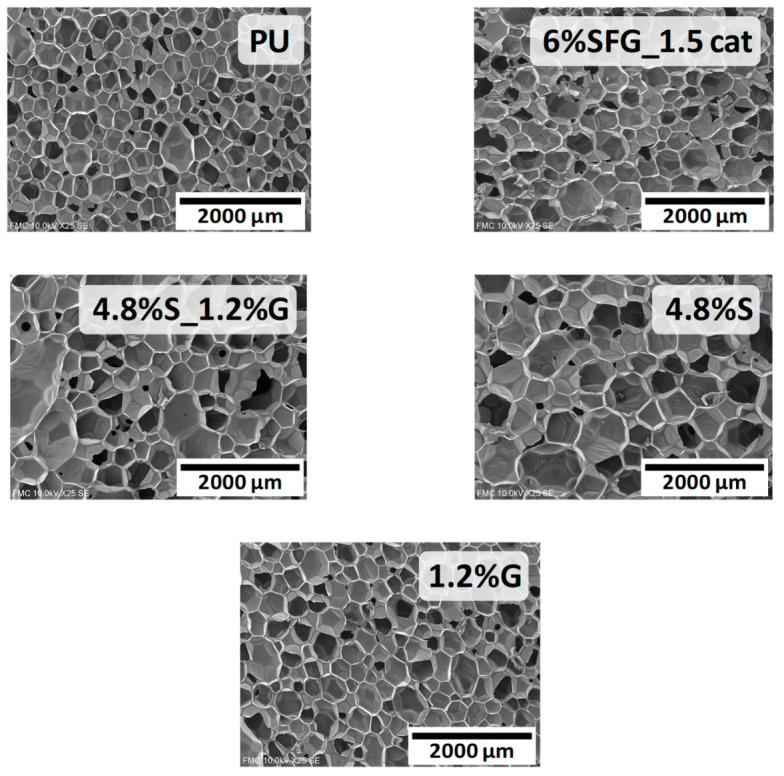
SEM micrographs in the growing plane of the RPU materials: the reference foam, the optimized foam with 6%wt SFG, the foam with only silane-modified nanosepiolites (S), the foam with only graphite (G), and the foam with S and G added separately.

In this final part, the characteristics of the optimized foam with 6 wt% SFG (4.8 wt% S functionalized with 1.2 wt% G) are compared with those of the foams containing only silane-modified nanosepiolites (4.8 wt% S), only graphite (1.2 wt% G), or silane-modified nanosepiolites and graphite added separately (4.8 wt% S and 1.2 wt% G), with the aim of demonstrating whether a synergistic effect produced by the functionalization of nanosepiolites with graphite exists. The densities, cellular structure characteristics and thermal conductivity of these RPU foams are shown in Table 8, and the cellular structure observed by SEM micrographs is displayed in Figure 11.

All of the materials with particles increase the density with respect to the reference foam; however, the optimized foam with 6 wt% SFG displays the smallest density increase at about 1.5 kg/m^3^. Regarding the OC values, the material containing only graphite hardly increases the open cell content compared with the unfilled material, whereas the material with only nanosepiolites reaches the highest open cell content at around 47%. This may be due to the large difference in the amount of nanosepiolites versus graphite (4.8 wt% vs. 1.2 wt%, respectively) in these samples, which may have permitted the agglomeration of nanosepiolites, thus promoting the opening of the cell walls. The same effect has been previously observed by the authors for RPU foams containing 5 wt% nanoclays and nanosilicas [38]. In addition, the combination of 4.8 wt% nanosepiolites and 1.2 wt% graphite produces an increase in the OC of around 25%, higher than that in the foam containing SFG, which is very low (only 8%). Considering the cellular structure, the foam with only graphite has a similar cell size and anisotropy to that of the unfilled material; however, the cellular structure is more heterogeneous (with high NSD). On the other hand, the foams containing only nanosepiolites and the combination of nanosepiolites and graphite show a large increase in cell size and a heterogeneous cellular structure (high NSD), whereas the incorporation of SFG clearly reduces the deterioration of cellular structure. All the samples are isotropic, with a similar anisotropy ratio, although this anisotropy value is reduced for the foam containing SFG.

Table 8 shows that the thermal conductivity value is increased for the foams containing reinforcements compared with that of the unfilled foam. The main reason for these results is a rise in foam density of up to 3 kg/m^3^ when the particles are included in the formulation (SFG, S and G), which raises the conduction through the solid phase, and thus the thermal conductivity. In addition to this, the samples with graphite (6%SFG_1.5cat, 4.8%S_1.2%G and 1.2%G) have increased conduction though the solid phase, because graphite particles can form a continuous conductive network [50]. In the case of the sample without graphite (4.8 %S), the thermal conductivity increases because of its high open cell content and its increased cell size, which provokes a considerable increase in heat transfer by radiation.

**Figure 12:**
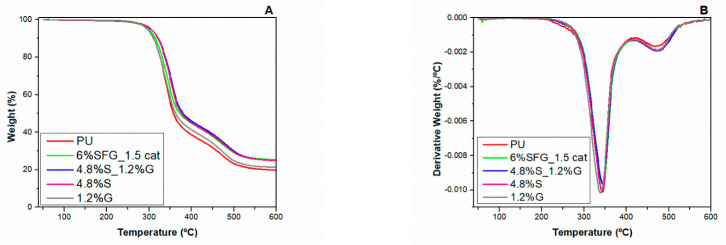
(**A**) TGA and (**B**) DTG curves for the RPU foams: the reference foam, the optimized foam with 6 wt% SFG, the foam with only silane-modified nanosepiolites (S), the foam with only graphite (G), and the foam with S and G added separately.

The TGA results of the different foam systems are presented in Figure 12 and the data are collected in Table 9. In general, the thermal stability is enhanced for all the foams with particles. The material containing 1.2 wt% graphite presents a thermal stability slightly above that of the reference foam, reaching a final loss mass of around 1.7%. However, the thermal stability of the rest of the samples with particles is much larger than that of the reference, reaching a final loss mass of around 5%. Thus, higher contents of nanosepiolite particles in the foams generate a greater effect on thermal stability than the presence of graphite. However, the use of SFG gives even better results, because the optimized sample with 6 wt% SFG presents the best thermal stability in the two degradation stages, achieving the highest percentage of loss mass (25.30% versus 19.76% of the reference). This is due to the synergystic effect of both types of particles. On the one hand, nanosepiolites act as a thermal insulator because their crystal structure is not modified; consequently, these particles remain in the char, increasing the residue mass [22,23]. In addition, nanosepiolites act as a transport barrier to the volatile gases formed by polyurethane decomposition [22,23]. On the other hand, the layered structure of graphite confers high thermal stability, as graphite char prevents the spread of flames in a fire situation [51]. Considering the overall results, the foam with 6 wt% of functionalized particles shows the best both thermal stability and cellular structure characteristics in comparison to the foams containing only nanosepiolites, only graphite, or nanosepiolites and graphite added separately.

## 4. Conclusions

The use of a promising flame retardant (FR) consisting of 80 wt% silane-modified nanosepiolites functionalized with 20 wt% graphite (SFG) on a water-blown rigid polyurethane (RPU) foam has been studied. Firstly, the effect of different amounts of SFG (0, 2, 4 and 6 wt%) on density, microstructure and thermal stability of the RPU foam is evaluated. These results indicate that the optimum amount of SFG in the PU material is 6 wt%, because although the thermal stability is very similar to that of the reference material, the residues reached in each step of degradation are higher. However, the cellular structure of this foam is clearly deteriorated with respect to the reference material without SFG, due to the increase in the cell size and the open cell content. In addition, the FTIR results show that SFG particles reduce both isocyanate consumption and the polymerization reaction, which affords urethane products.

The effect of the optimum SFG content (6 wt%) on the foam reactions are corrected by increasing a DMCHA catalyst (1.5 ppw) in the foam formulation in order to promote both the consumption of isocyanate and the gelling reaction. This strategy allows improvement of the cellular structure while at the same time maintaining the enhancement of thermal stability for the material with 6 wt% SFG. Moreover, the foam with 6 wt% SFG and 1.5 ppw DMCHA catalyst shows better cellular structure, thermal stability and thermal conductivity in comparison to the corresponding foams containing only silane-modified nanosepiolites (S), only graphite (G), or S and G added separately. In conclusion, the best performance is clearly achieved with the inclusion of SFG in RPU foams.

## Figures and Tables

**Table 1:** Formulations of RPU foams with different amount of SFG flame retardant, from 0 wt% to 6 wt%.

Samples	Polyol Component					Isocyanate Component
	SFG [wt%]	Polyol [ppw]	Surfactant [ppw]	Catalyst [ppw]	Blowing Agent (Water) [ppw]	Isocyanate Index
PU-Reference	**0**	100	1	1	6	120
2 wt% SFG	**2**	100	1	1	6	120
4 wt% SFG	**4**	100	1	1	6	120
6 wt% SFG	**6**	100	1	1	6	120

**Table 2:** Formulations of RPU foams with 6 wt% SFG when modifying the catalyst content.

Samples	Polyol Component					Isocyanate Component
	SFG [wt%]	Polyol [ppw]	Surfactant [ppw]	Catalyst [ppw]	Blowing Agent (Water) [ppw]	Isocyanate Index
PU-Reference	**0**	100	1	**1**	6	120
6 wt% SFG_1 ppw cat	**6**	100	1	**1**	6	120
6 wt% SFG_1.25 ppw cat	**6**	100	1	**1.25**	6	120
6 wt% SFG_1.5 ppw cat	**6**	100	1	**1.5**	6	120

**Table 3:** Formulations of the following materials: reference foam without FR, foam with 6 wt% SFG and the optimum amount of catalyst, foam with 4.8 wt% nanosepiolites (S), foam with 1.2 wt% graphite (G), and foam with 4.8 wt% nanosepiolites (S) and 1.2 wt% graphite (G) added in a different manner (particles without functionalization).

Samples	Polyol Component							Isocyanate Component
	SFG [wt%]	S [wt%]	G [wt%]	Polyol [ppw]	Surfactant [ppw]	Catalyst [ppw]	Blowing Agent (Water) [ppw]	Isocyanate Index
PU-Reference	0	0	0	100	1	1	6	120
6 wt% SFG_1.25 ppw cat	**6**	0	0	100	1	1.25	6	120
4.8 wt% S_1.2 wt% G	0	**4.8**	**1.2**	100	1	1.25	6	120
4.8 wt% S	0	**4.8**	0	100	1	1.25	6	120
1.2 wt% G	0	0	**1.2**	100	1	1.25	6	120

**Table 4:** Main cellular structure characteristics and physical properties of the manufactured RPU foams with different amounts of SFG (0, 2, 4, 6 wt%): density, open cell content (OC), mean cell size (Φ3D), normalized standard deviation (NSD) and anisotropy ratio (AR).

Samples	Density (kg/m^3^)	OC (%)	Φ3D (μm)	NSD	AR
PU-Reference	27.0 ± 0.1	2.7 ± 1.0	340 ± 90	0.26	1.1 ± 0.2
2 wt% SFG	29.1 ± 0.3	6.5 ± 0.9	390 ± 100	0.24	1.0 ± 0.2
4 wt% SFG	29.9 ± 0.3	12.9 ± 1.0	390 ± 100	0.24	1.0 ± 0.2
6 wt% SFG	31.8 ± 0.4	17.6 ± 1.2	500 ± 220	0.44	1.2 ± 0.3

**Table 5:** The viscosities of pure polyol and the mixtures of SFG dispersed in polyol.

Samples	Viscosity at 25 °C (mPa·s)
Polyol	510 ± 20
Polyol/2 wt% SFG	6300 ± 50
Polyol/4 wt% SFG	8280 ± 60
Polyol/6 wt% SFG	10,900 ± 100

**Table 6:** TGA and DTG results for RPU foams containing SFG from 0 to 6 wt%.

Samples	1st Step				2nd Step		
	T5 (°C)	Tmax1 (°C)	DTG max1 (%/°C)	Residue (%)	Tmax2 (°C)	DTG max2 (%/°C)	Residue (%)
PU-Reference	295.4 ± 0.0	341.9 ± 0.6	−0.0109	35.9 ± 0.2	474.1 ± 0.0	−0.0018	19.8 ± 0.8
2 wt% SFG	292.3 ± 0.1	341.0 ± 0.2	−0.0098	39.7 ± 0.2	472.6 ± 0.4	−0.0018	22.7 ± 0.1
4 wt% SFG	293.7 ± 0.7	343.0 ± 0.1	−0.0098	40.8 ± 0.2	472.7 ± 1.0	−0.0018	24.4 ± 0.4
6 wt% SFG	288.4 ± 1.4	343.1 ± 0.1	−0.0100	41.1 ± 0.3	474.5 ± 0.0	−0.0016	26.1 ± 0.3

**Table 7:** Density and primary cellular structure parameters of the RPU materials with 6 wt% SFG and different DMCHA catalyst contents (1, 1.25 and 1.5 ppw): density, open cell content (OC), mean cell size (Φ3D), normalized standard deviation (NSD) and anisotropy ratio (AR).

Samples	Density (kg/m^3^)	OC (%)	Φ3D (μm)	NSD	AR
6 wt% SFG_1 ppw cat	31.8 ± 0.4	17.6 ± 1.2	500 ± 220	0.44	1.2 ± 0.3
6 wt% SFG_1.25 ppw cat	30.7 ± 0.3	10.6± 1.4	400 ± 140	0.35	1.0 ± 0.2
6 wt% SFG_1.5 ppw cat	28.7 ± 0.6	7.9 ± 1.0	440 ± 160	0.36	0.9 ± 0.2

**Table 8:** Density and main cellular structure characteristics for the reference foam, the optimized foam with 6%wt SFG, the foam with only silane-modified nanosepiolites (S), the foam with only graphite (G), and the foam with S and G added separately: density, open cell content (OC), mean cell size (Φ3D), normalized standard deviation (NSD), anisotropy ratio (AR) and thermal conductivity.

Samples	Density (kg/m^3^)	OC (%)	Φ3D (μm)	NSD	AR	Thermal Conductivity (mW/mK)
PU-Reference	27.0 ± 0.1	2.7 ± 1.0	340 ± 90	0.26	1.1 ± 0.2	36.9 ± 0.2
6 wt% SFG_1.5 ppw cat	28.7 ± 0.6	7.9 ± 1.0	440 ± 160	0.36	0.9 ± 0.2	38.2 ± 0.3
4.8 wt% S_1.2 wt% G	30.5 ± 0.5	25.4 ± 3.3	540 ± 200	0.37	1.0 ± 0.3	39.1 ± 0.2
4.8 wt% S	30.0 ± 0.9	47.0 ± 2.2	590 ± 180	0.31	1.0 ± 0.2	38.8 ± 0.2
1.2 wt% G	29.3 ± 0.4	4.2 ± 1.9	360 ± 130	0.37	1.2 ± 0.3	38.4 ± 0.1

**Table 9:** TGA and DTG results for RPU foams.

Samples	First Step				Second Step		
	T5 (°C)	Tmax1 (°C)	DTG max1 (%/°C)	Residue (%)	Tmax2 (°C)	DTG max2 (%/°C)	Residue (%)
PU-Reference	295.4 ± 0.0	341.9 ± 0.6	−0.0109	35.9 ± 0.2	474.1 ± 0.0	−0.0018	19.8 ± 0.8
6 wt% SFG_1.5 ppw cat	297.8 ± 0.7	344.0 ± 0.1	−0.0094	42.5 ± 0.1	473.8 ± 0.3	−0.0019	25.3 ± 0.0
4.8 wt% S_1.2 wt% G	295.3 ± 0.0	344.4 ± 0.8	−0.0095	42.2 ± 0.2	475.0 ± 0.6	−0.0019	24.7 ± 0.3
4.8 wt% S	295.6 ± 0.1	342.7 ± 0.4	−0.0098	42.4 ± 0.4	473.8 ± 0.5	−0.0019	24.8 ± 0.9
1.2 wt% G	294.6 ± 1.3	338.8 ± 0.2	−0.0105	39.2 ± 0.1	474.8 ± 0.3	−0.0017	21.5 ± 0.5

## Data Availability

Not applicable.
